# Health economics: the start of clinical freedom

**DOI:** 10.1186/1472-6963-10-183

**Published:** 2010-06-28

**Authors:** José Antonio Sacristán, María Costi, Amparo Valladares, Tatiana Dilla

**Affiliations:** 1 Clinical Research Department. Eli Lilly and Company, Avenida de la Industria 30, Alcobendas, 28108 Madrid, Spain

## Abstract

**Background:**

Since Professor Hampton announced the death of clinical freedom in 1983, the increasing influence of Evidence-based Medicine and Health Technology Assessment has contributed to augment the feeling that clinicians have a secondary role in the therapeutic decision-making process.

**Discussion:**

This article constitutes a reflection on how clinicians may use the results of economic evaluations in their daily clinical practice, making decisions about cost-effectiveness on a case by case basis, and addressing both the patient's and society's needs. To that end, some illustrating examples are taken from the literature to show there are factors with great impact on cost-effectiveness results that can be easily identified and modified by clinicians.

**Summary:**

The evolution of the discipline and the trend towards a tailored therapy suggest that health economics is not the end of clinical freedom but the start of it.

## Background

Twenty five years ago Professor Hampton announced the death of clinical freedom [1]. He was then ahead of his time, when he reflected on the need of taking into account cost of interventions and evaluation techniques in the clinician's daily routine. Since Professor Hampton's paper, the increasing influence of Evidence-based Medicine (EBM) and Health Technology Assessment, has contributed to augment the feeling that clinicians play a secondary role in the therapeutic decision-making process.

In a rather simplistic way, one can distinguish an individual-patient ethic of effectiveness, based on providing each individual patient with the best available alternative, regardless of cost, and a population-health ethic of efficiency, based on providing the population with the best option according to available resources [2], but either way, it seems that treatment decisions increasingly depend on the results of meta-analyses, systematic reviews, and economic evaluations conducted by organisations such as the Cochrane Collaboration or NICE, to give but two very influential examples.

However, it would be a mistake to consider that doctor's new role is limited to automatically applying an evidence-based knowledge, or the results of the economic evaluations. In a patient-centered health care system it is just as harmful for clinicians not to take EBM or cost-effectiveness (CE) into consideration as it is for EBM and CE not to take doctors into consideration.

In the early days of CE analyses, it was very common to see generalizations and simplifications about the superiority or inferiority of one specific technology in comparison with other alternatives [3]. Fortunately, it is now increasingly common to see more sophisticated evaluations that try to take into account the full complexity and nuances of real clinical practice, analysing the different results in different subgroups of patients. This evolution to ensure that economic evaluations adapt their conclusions to patient subpopulations can be considered as one step forward in the "tailored therapy" concept. In general, health interventions are not efficient or inefficient in themselves but their efficiency depends on how they are used in each patient. Doctors are who finally decide how to use a certain technology in every specific patient. The efficacy and efficiency are usually obtained through quantitative methods (i.e. clinical trials and decision analytic models) that provide the best evidence for the average patient. But the best decision for the average patient is not necessarily the best decision for every single patient.

CE results should be considered as the starting point for doctors to decide what the "best" alternative in every case is, after taking into account all the relevant information of each individual patient. This article constitutes a reflection on how clinicians may use the results of economic evaluations of health interventions in their daily clinical practice, making decisions about cost-effectiveness on a case by case basis, and addressing both the patient's and society's needs. To that end, some illustrating examples are taken from the literature.

## Discussion

Results of cost-effectiveness analysis can be influenced by many factors. Some of them, such as the time horizon of the analysis or perspective of the analysis, are inherent to the characteristics of health economic models. However, others factors, such as the subgroup of patients for whom the intervention is most beneficial, the potential alternative treatments, or the best way to measure the outcome of a particular intervention, can be easily identified by clinicians.

The importance of subgroup analyses can be illustrated with the example of a CE study of cholesterol-lowering therapies analysed according to selected patient characteristics [4]. In the study women and men aged 35 to 84 years with high low-density lipoprotein cholesterol (LDLC) levels were divided into different risk subgroups according to age, sex, and the presence or absence of four coronary heart disease risk factors (smoking status, blood pressure, LDLC level, and high-density lipoprotein cholesterol level) for the economic evaluation. The results showed that incremental cost-effectiveness ratios (ICERs) for primary prevention with a statin compared with dietary management ranged from $54,000 per QALY to $1,400,000 per QALY, depending on risk subgroup characteristics. So, there are subpopulations for whom this intervention is not cost-effective because their corresponding CE ratios far exceed any international threshold, but it is also possible to identify the optimal subpopulation for whom the treatment meets the benchmark adopted by decision-makers. Identifying these patient subgroups for whom the technology might be particularly clinically and cost-effective, is a clear achievement in the economic evaluation but it must be translated into a more individualized therapy at the doctor's office. In this context, clinicians play a decisive role targeting the patient subgroups that will most benefit from a particular intervention, thus achieving a more efficient use.

The results of an economic evaluation of a health intervention may also vary depending on the choice of comparator [5]. An economic analysis performed on pegabtanib (indicated for age-related macular degeneration) showed how ICERs varied according to the comparator selected [6]. The incremental cost per QALY in patients receiving pegaptanib compared with those receiving photodynamic therapy with verteporfin was $49,052 and $59,039 for patients receiving pegaptanib versus standard of care. If a decision was to be made based strictly on US efficiency limits (i.e. $50,000), pegaptanib would only be considered a cost-effective option vs. photodynamic therapy with verteporfin. For this reason, when doctors are considering using a particular therapeutic intervention, they must think carefully about the alternative treatment for that same patient, since this information will influence treatment efficiency.

With regard to the outcome measures, efficacy usually differs from effectiveness. Factors such as adherence rate, dose adjustment, length of treatment, use of concomitant medication etc. may explain the difference. The CE analysis of angiotensin-converting-enzyme inhibitor therapy for diabetic nephropathy confirms the importance of treatment adherence in clinical practice, since ICERs varied substantially with different adherence rates [7]. Changes in the compliance rate from 65% to 51% could result in a swing in CE from $4,091 per QALY to $1,176,738 per QALY. Clinicians play a very active role in improving patients' adherence to treatment, contributing to closing the gap between efficacy and effectiveness and consequently improving patients' outcomes and the cost-effectiveness of interventions. Doctors could also contribute towards more efficient medicine by prescribing formulations that facilitate therapeutic compliance only in patients who have shown poor treatment adherence.

In addition, the decision-making process is not based solely on efficiency criteria since social values judgments are applied to enhance fairness and reduce health inequalities. Although the growing importance of these social value judgements may be acknowledged, we must not neglect "individual patient values*"*. Accounting for patient preferences and quality-of-life concerns may shift the balance of CEA results of particular health interventions. For example, incorporating patient-derived preferences regarding complications and treatments of diabetes improved ICERs for intensive glucose control in older type 2 diabetic patients [8]. However, in daily practice, it is doctors who may incorporate these factors in their clinical decisions. Doctors make therapeutic decisions based on the available information for average patients, but they are aware that, sometimes, individual patient values may modify these decisions. For example, patients may refuse a cancer treatment that is backed by clinical trial evidence and has positive cost-effectiveness, simply because they are reluctant to suffer the side effects of the therapy, or they may prefer avoiding an inconvenient preventive intervention because they are willing to take the bigger risk of getting the disease, or suffering the complication that the intervention sought to prevent. Just as the scientific value judgements of organisations such as NICE should be "individualized" for each patient by the doctor, social value judgements should be put within the perspective of individual value judgements.

Some ethical challenges may arise from the clinical implementation of these reflections, in relation to the principle of distributive justice: is the practice of patient-centered care compatible with a fair and equal allocation of health care resources? [9]. Ensuring a better use of available health care resources by targeting therapies to the patients' subgroups that can benefit most leads to improvements in its cost-effectiveness at individual level and this shouldn't cause ethical concerns, but quite the opposite.

In conclusion, in the era of individualized medicine, decisions about CE should also be made on a case by case basis. Experts in methodology and decision makers should focus on evaluating interventions, whereas clinicians are the professionals who evaluate patients. Society must establish social value judgments if resources are to be distributed with efficiency and equity, but only doctors can take individual value judgments into account, and these values must have special relevance in the times of evidence-based medicine and efficient medicine. In these times, physicians must be aware of their crucial role choosing the best intervention for every patient, considering that the efficacy and efficiency are abstract concepts that become real only when the intervention is used in the individual patient in the daily clinical practice. For all the reasons highlighted above, we believe health economics shouldn't be considered the end of clinical freedom, but the start of it.

## Summary

• The evolution of the discipline of economic evaluation in health care shows that clinicians must play a decisive role if we are to achieve the target of having a more efficient health service.

• Interventions are not efficient or inefficient *per se*, their efficiency is determined when they are used in clinical practice.

• Even if the practice of personalized medicine may seem challenged by ethical issues, social and individual value judgments are not mutually exclusive.

• Health economics is not the end of clinical freedom but the start of it. Doctors take up a central position in the health care system and they may contribute to finding the right balance between clinical freedom and social responsibility.

## Competing interests

All authors are full-time employees of Eli Lilly and Company.

## Authors' contributions

This paper arose out of discussions between the authors about the rationale of decision-making in the health field. JAS contributed to conception, design and first draft of the manuscript. AV and MC managed the literature searches and analyses. TD contributed to coordination and critical review of the manuscript. All authors were involved in discussions about the concepts in the paper, together with its format, and have given final approval of the version to be published. JAS is guarantor of the manuscript.

## Pre-publication history

The pre-publication history for this paper can be accessed here:

http://www.biomedcentral.com/1472-6963/10/183/prepub
